# Comparison of sand fly trapping approaches for vector surveillance of *Leishmania* and *Bartonella* species in ecologically distinct, endemic regions of Peru

**DOI:** 10.1371/journal.pntd.0009517

**Published:** 2021-07-14

**Authors:** Victor O. Zorrilla, Marisa E. Lozano, Liz J. Espada, Michael Kosoy, Clifton McKee, Hugo O. Valdivia, Heriberto Arevalo, Mario Troyes, Craig A. Stoops, Michael L. Fisher, Gissella M. Vásquez

**Affiliations:** 1 Department of Entomology, U.S. Naval Medical Research Unit No.6, Bellavista, Peru; 2 Vysnova Partners, Lima, Peru; 3 KB One Health LLC, Fort Collins, Colorado, United States of America; 4 Graduate Degree Program in Ecology, Colorado State University, Fort Collins, Colorado, United States of America; 5 Department of Biology, Colorado State University, Fort Collins, Colorado, United States of America; 6 Department of Epidemiology, Johns Hopkins Bloomberg School of Public Health, Baltimore, Maryland, United States of America; 7 Department of Parasitology, U.S. Naval Medical Research Unit No.6, Bellavista, Peru; 8 Laboratorio Referencial de Salud, Tarapoto, Direccion Regional de Salud San Martin, Peru; 9 Direccion de Salud DISA Jaen, Cajamarca, Peru; RTI International, UNITED STATES

## Abstract

**Background:**

In Peru, the information regarding sand fly vectors of leishmaniasis and bartonellosis in the Amazon region is limited. In this study, we carried out sand fly collections in Peruvian lowland and highland jungle areas using different trap type configurations and screened them for *Leishmania* and *Bartonella* DNA.

**Methodology/Principal findings:**

Phlebotomine sand flies were collected in Peruvian Amazon jungle and inter Andean regions using CDC light trap, UV and color LED traps, Mosquito Magnet trap, BG Sentinel trap, and a Shannon trap placed outside the houses. *Leishmania* spp. screening was performed by kDNA PCR and confirmed by a nested cytochrome B gene (*cytB*) PCR. *Bartonella* spp. screening was performed by ITS PCR and confirmed by citrate synthase gene (*gltA*). The PCR amplicons were sequenced to identify *Leishmania* and *Bartonella* species.

UV and Blue LED traps collected the highest average number of sand flies per hour in low jungle; UV, Mosquito Magnet and Shannon traps in high jungle; and Mosquito Magnet in inter Andean region. *Leishmania guyanensis* in *Lutzomyia carrerai carrerai* and *L*. *naiffi* in *Lu*. *hirsuta hirsuta* were identified based on *cytB* sequencing. *Bartonella* spp. related to *Bartonella bacilliformis* in *Lu*. *whitmani*, *Lu*. *nevesi*, *Lu*. *hirsuta hirsuta* and *Lu*. *sherlocki*, and a *Bartonella* sp. related to *Candidatus* B. rondoniensis in *Lu*. *nevesi* and *Lu*. *maranonensis* were identified based on *gltA* gene sequencing.

**Conclusions/Significance:**

UV, Blue LED, Mosquito Magnet and Shannon traps were more efficient than the BG-Sentinel, Green, and Red LED traps. This is the first report of *L*. *naiffi* and of two genotypes of *Bartonella* spp. related to *B*. *bacilliformis* and *Candidatus* B. rondoniensis infecting sand fly species from the Amazon region in Peru.

## Author summary

Leishmaniasis and human bartonellosis, two neglected tropical diseases, are endemic in Peruvian Andean regions. However, no human bartonellosis cases has been reported in the Amazon jungle where leishmaniasis has high prevalence. The improvement of sand fly trapping methods and identification of putative *Leishmania* and *Bartonella* vectors across Andean and Amazonian regions are important steps towards the design of better control strategies and reducing the risk of transmission in endemic areas. In this paper the authors explored the effectiveness of different trap types, based in LED colors and odor attractants, for sand fly collections in endemic areas of leishmaniasis and bartonellosis. Also, we reported for the first time the detection of *Bartonella* DNA in sand fly species from the Peruvian Amazon jungle, and the first record in Peru of *L*. *(V*.*) naiffi* infecting *Lu*. *hirsuta hirsuta*. The information provided by this manuscript will serve as a baseline for future surveillance and intervention studies about sand fly species as potential vectors of *Leishmania* and *Bartonella* in highly endemic areas.

## Introduction

Phlebotomine sand flies are small insects of crepuscular and nocturnal activity with weak flight capabilities, distributed in tropical and sub-tropical regions around the world. Female sand flies must feed on blood to complete oogenesis and lay eggs, and obtain blood meals from a large variety of vertebrates, including humans [1]. Due to this broad host range, some phlebotomine species may transmit pathogens to a vertebrate host, such as parasites of the genus *Leishmania*, bacteria of the genus *Bartonella*, and some arboviruses [2,3]. Phlebotomine sand flies have an important role in the epidemiology of these diseases; monitoring and control of sand flies are, therefore, of high priority [4].

The leishmaniases are a group of neglected tropical diseases endemic in 98 countries worldwide with 0.9 to 1.6 million new cases per year, between 20,000 and 30,000 deaths, and 350 million people at risk of infection [5]. In Peru, more than 4,000 new cases of cutaneous and mucocutaneous leishmaniasis are reported yearly; no visceral leishmaniasis cases have been reported to date [6]. Human bartonellosis, also called Carrion’s disease or Oroya fever, is caused by *Bartonella bacilliformis* and is a severe, debilitating illness with a mortality rate of over 80% in untreated individuals [7]. The initial infection is characterized by fever and severe anemia; the secondary infections in this phase are associated with transitory immune system depression. After a variable time of acute infection, the presence of vascularized skin warts (“verruga peruana”, which is considered the chronic presentation of Carrion’s disease) is frequent [8]. This neglected disease is limited to Peru, Ecuador, and Colombia, with higher prevalence in Peru where new endemic areas have been recently reported [7,9]; no cases have been reported during the last two decades in the latter two countries. Other *Bartonella* species can cause long-lasting intra-erythrocytic infections in their mammalian reservoirs and are transmitted by other arthropod species [10–12]. In Peru, leishmaniasis and human bartonellosis are endemic in native communities located in the Andean region, between 1,000 and 3,200 m altitude [13,14] where *Lu*. *verrucarum* and *Lu*. *peruensis* (Diptera: Psychodidae: Phlebotominae) are considered the main vectors of both diseases [15,16]. In contrast, reports of human bartonellosis cases in the Peruvian Amazon jungle, where leishmaniasis has the highest prevalence, are limited to some high jungle valleys in the northeastern and central regions [17,18]. However, there is limited or no information regarding sand fly vectors of human bartonellosis in the Peruvian Amazon region [19].

In order to perform efficient surveillance of pathogens carried by phlebotomine sand flies, we need to understand which trapping methods are most successful for catching sand flies. Phlebotomine sand flies are generally attracted by artificial lights of different intensity and color, CO_2_, and other chemical attractants recommended for use in hematophagous insect and vector surveillance [3,20,21]. CDC light traps with incandescent lights are the most widely used [2], while the Shannon trap with protected human bait is the most effective method for capture of anthropophilic sand fly species [22–24]. In Peru, CDC light traps, Shannon traps, and resting site collection have been used for decades in sand fly vector surveillance in Andean regions, and high and low jungle areas [13,15,22,25,26]. Although the Shannon trap and protected human bait are the most effective methods for anthropophilic sand fly collection and are used to obtain information on man-biting behavior, their use is limited due to the increased risk for pathogen infection, including leishmaniasis, to human bait [27,28]. Also, the climatic or human factors (rain, wind, environmental modification) can influence the effectiveness of these trapping methods in different study sites [29], which demonstrates the importance of using a variety of trap types in studies of sand fly fauna. Additionally, the rapid spread of vector-borne diseases transmitted by sand flies to new endemic foci raises the need to implement more effective sand fly collection methods [7,21,30]. In addition to classic methods for sand fly collection, other trap types based on light emission or baited with chemical attractants, including Mini CDC LED traps, UV traps, BG-Sentinel and Mosquito Magnet, have been used in endemic areas of leishmaniasis. Interestingly, trap performance varied considerably across geographic regions [4,31–35], which could be linked to distinct sand fly behaviors and ecological niches as well as environmental factors. Thus, it is important to identify effective sand fly trapping methods in highly diverse endemic regions [36] such as the Peruvian Amazon.

Currently, more than 190 sand fly species have been identified in Peru, 80% of them in the Amazon region [16]. However, there are few studies where, in addition to characterizing sand fly species diversity, detection of *Leishmania* has been done, particularly in high and low rainforest areas where approximately 17 *Lutzomyia* species were found infected with *L*. *(V*.*) braziliensis*, *L*. (*V*.) *lainsoni*, *L*. (*V*.) *guyanensis* and *L*. (*Viannia*) spp. [16,37]. The detection of *Bartonella* DNA in sand flies has had limited success, with most studies conducted in inter-Andean regions where Carrion’s disease is endemic, principally in Cuzco and Ancash departments where two species, *Lu*. *peruensis* and *Lu*. *verrucarum*, were found infected with *B*. *bacilliformis* [38–40]. Epidemiological data suggested that *Lu*. *robusta* and *Lu*. *maranonensis* are potential vectors of Carrion’s disease in high jungle areas in the northeastern region [19]. Additionally, *Lu*. *maranonensis* was reported recently as a new potential vector of *B*. *bacilliformis* in Cutervo, Cajamarca, where this disease is endemic [41]. However, to date we do not have any reports about the transmission of Carrion’s disease in the Amazonian region, and if it may also co-circulate with *Leishmania* and be transmitted by leishmaniasis vectors.

In this study, we propose to evaluate for the first time in Peru different light and odor-baited trap types for catching phlebotomine sand flies, expecting to increase our understanding of the foraging behavior of sand fly species present in different ecological regions. The objective of this study was to evaluate different vector trapping methods for their efficacy in capturing sand flies and to identify potential sand fly vector species of two neglected diseases, leishmaniasis and bartonellosis, in different ecological sites in Peruvian lowland and highland jungle areas and inter-Andean regions. We hypothesized that there would be a significant variation in the response of sand flies to different trap types according to study site and sand fly fauna composition. Moreover, we screened female sand flies captured for both *Leishmania* and *Bartonella* DNA detection to identify potential vector species. Given the high sand fly diversity in selected study sites, we assumed that different *Leishmania* and *Bartonella* species are circulating in geographical regions where their presence has not been previously reported, especially in the Amazon region.

## Materials and methods

### Study design

In July 2014, and January and February 2015, multiple trap configurations were tested for their efficacy in collecting phlebotomine sand flies in four sites located in different ecological regions where leishmaniasis and/or human bartonellosis are endemic. We morphologically identified female and male sand flies, then tested non-engorged female sand flies (no visible blood meal in the sand fly gut) for molecular detection of *Bartonella* and *Leishmania* DNA. Phylogenetic analyses were performed on DNA sequences obtained.

### Study sites

**Fig 1:**
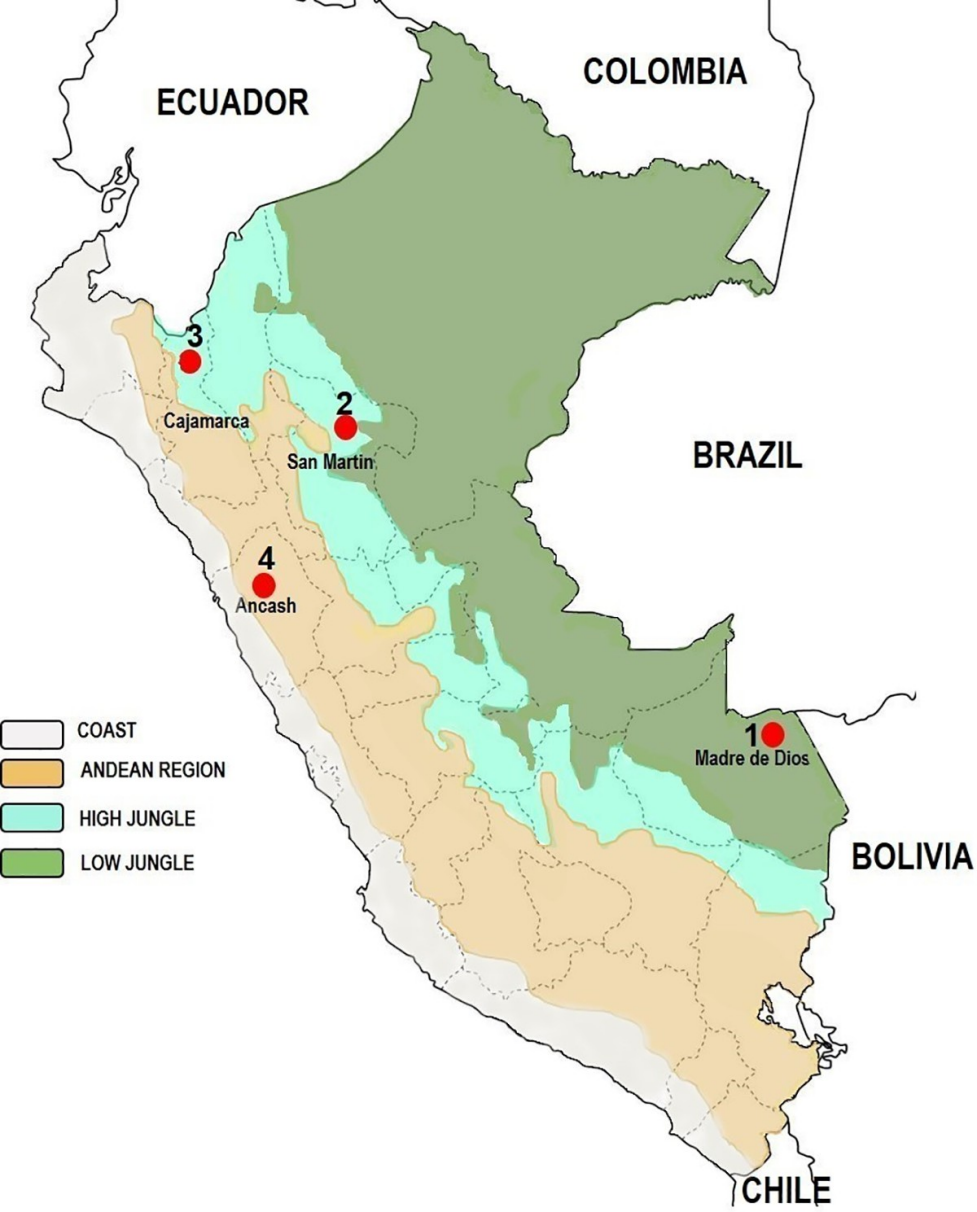
Study sites in three different ecological regions within Peru. The map was created using open data obtained from Wikipedia. URL: https://en.wikipedia.org/wiki/Geography_of_Peru#/media/File:Peru_veg_1970.png (public domain).

We carried out sand fly collections in Peruvian lowland and highland jungle areas, and one inter-Andean Valley (Fig 1). All collections were performed in peri- and extradomiciliary sites.

1. **Flor de Acre, Madre de Dios.** A lowland rainforest area located in Iberia district, province of Tahuamanu, Madre de Dios region (11°19’54.3”S, 69°36’20.6”W, 292 m.a.s.l.), near the Peru-Brazil-Bolivia triple border (southeastern Peru).
2. **Tarapoto, San Martin**. A highland jungle area located in the province of San Martin, San Martin region, northeastern Peru. Sand fly collection was conducted in three rural sites located approximately 5 kilometers from Tarapoto city, along the Shilcayo River: Bocatoma de Shilcayo water station (06° 27’39.3”S, 76° 21’01.8”W, 419 m.a.s.l.); Centro de Rescate Urku (06° 27’ 51.9”S, 76° 21’ 07.5”W, 415 m.a.s.l.), a protected natural area; and a small rural hostel Cordillera Escalera Lodge (06° 28’ 05.4”S, 76° 21’ 12.2”W, 403 m.a.s.l.).
3. **San Jose de Lourdes, Cajamarca**. This rural community is located in the Chinchipe Valley, highland jungle area, approximately 30 kilometers from the Peru-Ecuador border, in San José de Lourdes district, province of San Ignacio, Cajamarca region (05° 04’ 58.2" LS, 78° 55’ 10.2" LW, 1264 m.a.s.l.), northeastern Peru.
4. **Caraz, Ancash**. A rural community (Choquechaca**)** located in the district of Caraz, province of Huaylas, Ancash region, along the inter Andean Santa River valley (08° 59’12.0" LS, 77° 49’22.8" LW, 2159 m.a.s.l.).

Rainfall and high humidity are predominant in high and low jungle areas (Madre de Dios, San Martin and Cajamarca), the climate is tropical with an annual mean temperature over 26°C. The main activity of the population is agriculture and cattle breeding in Madre de Dios and San Martin, whereas in San Jose de Lourdes, Cajamarca, coffee, cacao and fruit crops are predominant. Caraz, Ancash, is a typical inter-Andean valley with dispersed housing in the rural area surrounded by crops (grasses, tubers, cereals) and livestock (sheep, cows, horses).

### Sand fly collection

**Fig 2:**
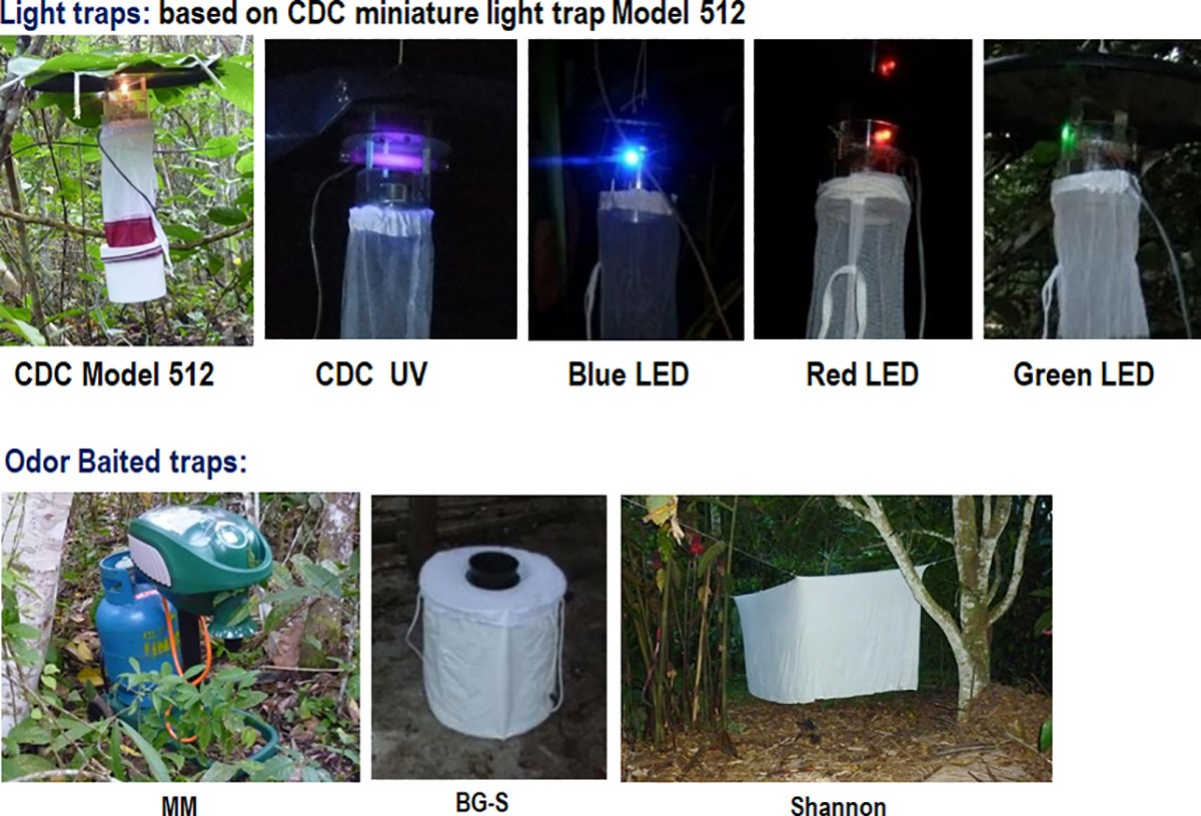
Different trap types used for sand fly collections. LED colors were adapted from the standard Mini CDC light trap Model 512 (CDC). MM: Mosquito Magnet Model Independence baited with CO_2_ and R-Octenol; BG-S: BioGents BG Sentinel baited with BG-Lure.

Trap locations within each village were selected based on the occurrence of human leishmaniasis and bartonellosis cases reported by the local Ministry of Health. A Latin square design was employed with traps being placed in the peridomicile and extradomicile (edge of the forest and inside the forest) environments and at least 50 meters apart to eliminate interference. No intradomiciliary sand fly collections were performed due some traps release CO_2_ or emit UV light. This study involved attracting sand flies using luminous traps and odor baited traps that run overnight (1800–0600 hours) (Fig 2) during two consecutive days in Madre de Dios, five days in San Martin, and four days in Cajamarca and Ancash, respectively.

#### Luminous traps

We used the Mini CDC light traps Model 512 (John W. Hocks Co., Gainesville, FL, USA) with a 6.3 W incandescent lamp (CDC, Atlanta, GA, USA); Blacklight UV traps (UV) Model 1212 (John W. Hocks Co.) with a 6.3 W black light (F4T5 BLB), and LED technology with Mini CDC light trap Model 512 modified locally to replace the incandescent lamp with blue, green, or red LED color bulbs [31,42]. All luminous traps were placed approximately one meter above ground level and operated overnight from 1800 to 0600 hours. The energy for light traps was provided by rechargeable 6 V gel batteries (Opalux, Lima-Peru).

#### Odor baited traps with chemical attractants

Two commercial traps were tested, BioGents Sentinel Trap (BG; BioGents, Regensburg, Germany) with BG Lure as the attractant; and Mosquito Magnet (MM; Woodstream Coroporation, Lancaster, PA, USA) Trap Model Independence MM3200B which is battery-operated and runs on R-Octenol and propane gas, which is catalytically converted to produce CO_2_, heat and water vapor, mimicking a vertebrate host and attracting biting insects [27,31,34]. These traps operated overnight from 1800 to 0600 hours.

#### Shannon trap with protected human bait

This trap was used as a complementary method for sand fly collection in some places and when the weather conditions allowed. Insects were collected with Shannon trap from 1800 to 2100 hours by one operator protected with a mosquito jacket acting as human bait and using a manual aspirator and head lantern to manually catch sand flies [22,24]. The Shannon trap collections did not use volunteers and were instead performed by experienced and highly trained NAMRU-6 entomology researchers. NAMRU-6 Occupational Health & Safety regulations for entomological specimen field collection were followed accordingly.

### Sand fly taxonomic identification

Sand fly specimens collected were counted and sorted by location, collection method, sex and collection date, stored in 1.5 mL microcentrifuge tubes with 70% ethanol, and transported to the Entomology Department at NAMRU-6 in Bellavista, Callao region. Specimens were identified morphologically using taxonomic keys and procedures developed by Young and Duncan (1994) [1] and Galati (2003) [43] with small modifications. Female specimens were processed with a modified protocol where the head and last two abdominal segments were separated and clarified with lactophenol (phenol:lactic acid 2:3) for two hours for taxonomic identification, while the rest of the body from each female sand fly was pooled (1–10 specimens per tube) in microcentrifuge tubes with 70% ethanol according to species, date, site and collection method, and stored at -20°C [37,44]. Male specimens were clarified in 20% KOH for 12–24 hours followed by lactophenol for 2 hours. Voucher specimens were mounted permanently on Euparal.

### Sand fly abundance and diversity analyses

Species abundance was calculated in Microsoft Excel 2013 using the Index of Species Abundance (ISA) [45] using the formula *ISA* = (a + R_j_)/k, where we established a rank for species abundance per site; “a” is the number of zero observations for each species in all sites multiplied by “c” which is the single largest rank in all the data set plus 1. The R_j_ value corresponds to the sum of ranks for a given species in all the sites whereas “k” corresponds to the number of sites. The resulting ISA values were converted into the Standardized Index of Species Abundance (SISA) using the formula *SISA* = (*c* − *ISA*)/(*c* − 1). We quantified the number of sand flies captured per hour over each day of trapping and cumulatively over all trapping days. Normality of data was assessed by the Kolmogorov-Smirnov test in Minitab 17.1.0. Since the data did not meet the normality requirement, we used a non-parametric test for data analysis. A Kruskal-Wallis test was performed to assess whether sand fly capture rates per hour varied significantly between trap types for each site on separate days. Pairwise Wilcoxon signed-rank tests corrected for multiple comparisons using a Benjamini-Hochberg procedure were used to identify the trap type that accounted for significant differences [46]. The total sand fly species richness was quantified for each study site and trap type. Shannon-Wiener diversity indices of species evenness for each study site and trap type were calculated using the equation $H=-\sum_{i=1}^{n}p\;\ln\;p$, where “p” represents the proportion of each species “n” that was collected (∑p = 1). The Hutcheson t-test was used to assess differences in Shannon diversity between trap types at each site [47]. All analyses were carried out in PAST v3.12 and R v4.0.2.

### Molecular screening of *Leishmania* and *Bartonella* DNA in sand flies

#### DNA extraction

Pools of non-engorged phlebotomine sand flies were air-dried to remove ethanol and ground with pellet-pestles in lysis buffer. Genomic DNA was extracted according to the manufacturer’s protocol using the DNeasy Blood & Tissue kit (QIAGEN, Valencia, CA). The final DNA was eluted in 50 μL of elution buffer, and frozen at -30°C. All PCR conditions for *Leishmania* and *Bartonella* screening are provided in S1 Text.

#### Molecular detection of *Leishmania* DNA

The initial screening for *Leishmania* was performed by PCR that targets *Leishmania* minicircle kinetoplast DNA (kDNA; approx. 700bp) conserved among species, using primers L.MC-1S and L.MC-1R as previously described [48] with some modifications on reagents concentration (S1 Text). Positive samples for kDNA PCR were confirmed by nested PCR targeting cytochrome *b* (*cytB*) [49,50]. Details of primers used are shown in Table 1. *L*. *(V*.*) braziliensis* and *L*. *(Leishmania) infantum* DNA were used as positive PCR controls whereas PCR mix without DNA was used as a negative control.

#### Molecular detection of *Bartonella* DNA

A PCR protocol that amplifies a segment between 400-600bp of the 16S–23S internal transcribed spacer (ITS) region was used for screening sand flies for *Bartonella* DNA [51]. Confirmation of positive samples was performed using a nested PCR protocol for *gltA*, the citrate synthase gene [52–54]. Both markers have been validated on a broad diversity of *Bartonella* species but have different advantages that work well in combination. ITS is highly sensitive for detection of *Bartonella* DNA, but sequence alignment and phylogenetic analysis are complicated by the many insertions and deletions present. In contrast, *gltA* is less sensitive but is more amenable to alignment and phylogenetic analysis. Furthermore, *gltA* is the most commonly used marker for molecular detection of *Bartonella* species, creating a large database for comparison [55]. Additional molecular markers, *rpoB*, the β subunit of bacterial RNA polymerase gene [56,57], and *ftsZ*, the filamenting temperature-sensitive mutant Z protein gene [58,59] were used to confirm ITS PCR and *gltA* PCR results (S1 Text). Details of primers used are shown in the Table 1. *Bartonella bacilliformis* DNA from culture (SANDI strain from Caraz, Ancash, Peru) [39] was used as a positive control and the PCR mix without DNA as a negative control. Both controls were included in each PCR run to evaluate the presence of appropriately sized amplicons and contamination, respectively. Only samples with PCR products that resulted in a correct amplicon size and a clear band were considered as positive for further DNA sequencing and phylogenetic analysis.

**Table 1:** Primer sequences for *Leishmania* and *Bartonella* DNA amplification from sand flies.

Locus	Primers		Primer 5’-sequence-3’	Size of PCR Product (bp)	PCR Protocol Reference
***Bartonella***					
16S–23S internal transcribed spacer	ITS	325S	CTTCAGATGATGATCCCAAGCCTTTTGGCG	400–600	[51]
		1100AS	GAACCGACGACCCCCTGCTTGCAAAGCA		
Citrate synthase gene	gltA	443F	GCTATGTCTGCATTCTATCA	767	[11]
		1210R	GATCYTCAATCATTTCTTTCCA		
		BhCS.781p	GGGGACCAGCTCATGGTGG	380–400	[52]
		BhCS.1137n	AAATGCAAAAAGAACAGTAAACA		
Filamenting temperature-sensitive mutant Z protein gene	ftsZ	BFP-1	ATTAATTCTGCAYCGGCCAGA	600	[58]
		BFP-2	ACVGADACACGAATAACACC		
		R83	ATATCGCGGAATTGAAGCC		[59]
		L83	CGCATAGAAGTATCATCCC		
RNA polymerase β subunit gene	rpoB	1350F	GGCAATCGTCGCGTTCGTTC	852	[56]
		2350R	CTACCCGATCACCAACATGC		
		1400F	CGCATTGGCTTACTTCGTATG		[57]
		2300R	GTAGACTGATTAGAACGCTG		
***Leishmania***					
kinetoplast DNA	L.MC	L.MC-1S	CTRGGGGTTGGTGTAAAATAG	700	[48]
		L.MC-1R	TWTGAACGGGRTTTCTG		
cytochrome b gene	L.cyt-B	L.cyt-AS	GCGGAGAGRARGAAAAGGC	1070–1080	[49]
		L.cyt-ASR	CCACTCATAAATATACTATA		
		L.cyt-S	GGTGTAGGTTTTAGTYTAGG	730–850	[48]
		L.cyt-R	CTACAATAAACAAATCATAATATRCAATT		

#### *Leishmania* and *Bartonella* DNA sequencing

The positive *Leishmania* and *Bartonella* PCR products were analyzed by electrophoresis on 2% agarose gel stained with GelRed (Biotium, Fremont, CA, USA). The PCR amplicons were purified using the Wizard SV Gel and PCR Clean-Up System (Promega, Madison, WI, USA) and sequenced along both DNA strands using the BigDye Terminator v3.1 Cycle Sequencing Kit (Applied Biosystems, Foster City, CA, USA) according to the manufacturer’s instructions. Samples were sequenced at the Entomology Laboratory at NAMRU-6 (Lima, Peru) using an Applied Biosystems 3130 XL Genetic Analyzer sequencer. Sequences were confirmed as being *Leishmania* or *Bartonella* DNA using the NCBI Basic Local Alignment Search Tool (BLAST; https://blast.ncbi.nlm.nih.gov/Blast.cgi) prior to phylogenetic analysis.

#### Phylogenetic analysis of *Leishmania* DNA sequences

The alignment of *cytB* gene sequences obtained from sand flies was made using the ClustalW method, and sequences were analyzed with MEGA (Molecular Evolutionary Genetics Analysis) program version X [60]. The evolutionary distances were computed using the Kimura 2-parameter method [61]. Phylogenetic trees were constructed with the maximum likelihood method with Tamura-Nei model [62] and bootstrap support values for nodes were determined with 1,000 replicates [63]. Datasets of *cytB Leishmania* spp. reference sequences were obtained from GenBank.

#### Phylogenetic analysis of *Bartonella* DNA sequences

*Bartonella gltA* sequences obtained from sand flies were aligned with reference sequences for named *Bartonella* species from GenBank using MAFFT v7 [64]⁠. The alignment was trimmed to a common length to eliminate poorly aligned positions using Phyutility v2.2 and Gblocks v0.91b [65,66]. Alignments were visually inspected for errors and manually corrected. A maximum likelihood tree was generated using RAxML v8 [67] using only the most closely matching reference sequences in the alignment. The GTRCAT model was used with 25 distinct rate categories and branch support was estimated with 1,000 bootstrap replicates.

## Results

### Trap type evaluation for sand fly collection

#### Flor de Acre, Madre de Dios

The CDC, blue, green and red LED, UV and Shannon traps were tested. A total of 2,328 sand fly specimens were collected edge of the forest, 913 (39.2%) females and 1,415 (60.8%) males belonging to two genera, *Lutzomyia* (35 species) and *Brumptomyia* (2 species). Abundance of species identified in all trap types used showed that *Lu*. *davisi* (SISA = 0.96), *Lu*. (*Trichophoromyia*) spp. (SISA = 0.91), *Lu*. *yucumensis* (SISA = 0.90), *Lu*. *whitmani* (SISA = 0.87) and *Lu*. *auraensis* (SISA = 0.80) were at the highest density (S1 Table). *Lutzomyia davisi* was the most abundant sand fly species in red LED trap (55.0%), *Lu*. *auraensis* was predominant in green LED and UV (70.5% and 29.6% respectively), *Lu*. *whitmani* in blue LED and CDC (34.6% and 24.6% respectively), and *Lu*. *yucumensis* was the predominant species in the sand fly collections with Shannon trap (55.7%).

**Fig 3:**
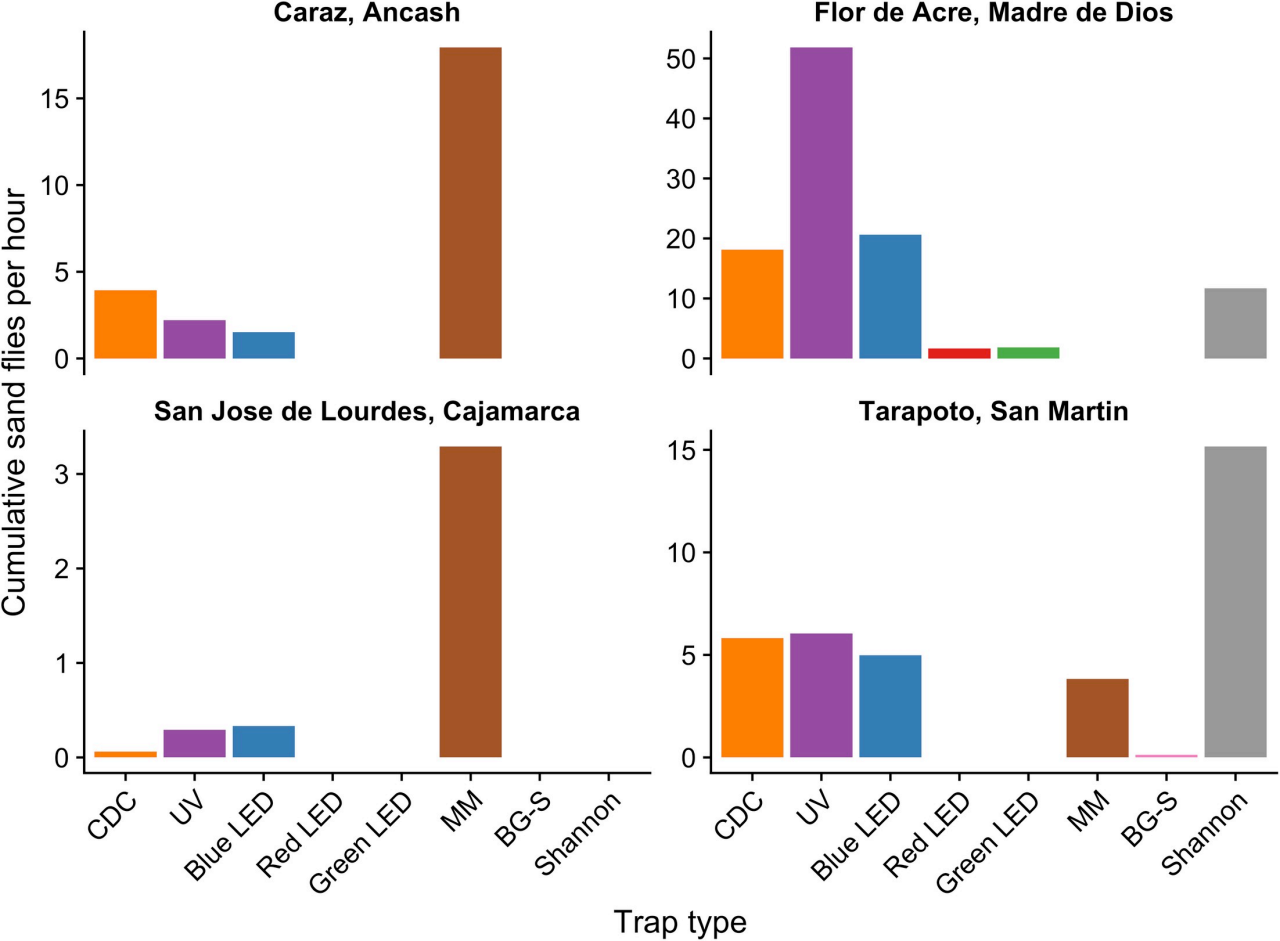
Comparison of cumulative sand flies per hour collected from 1800 to 0600 for a two-day period across trap types and study sites.

**Fig 4:**
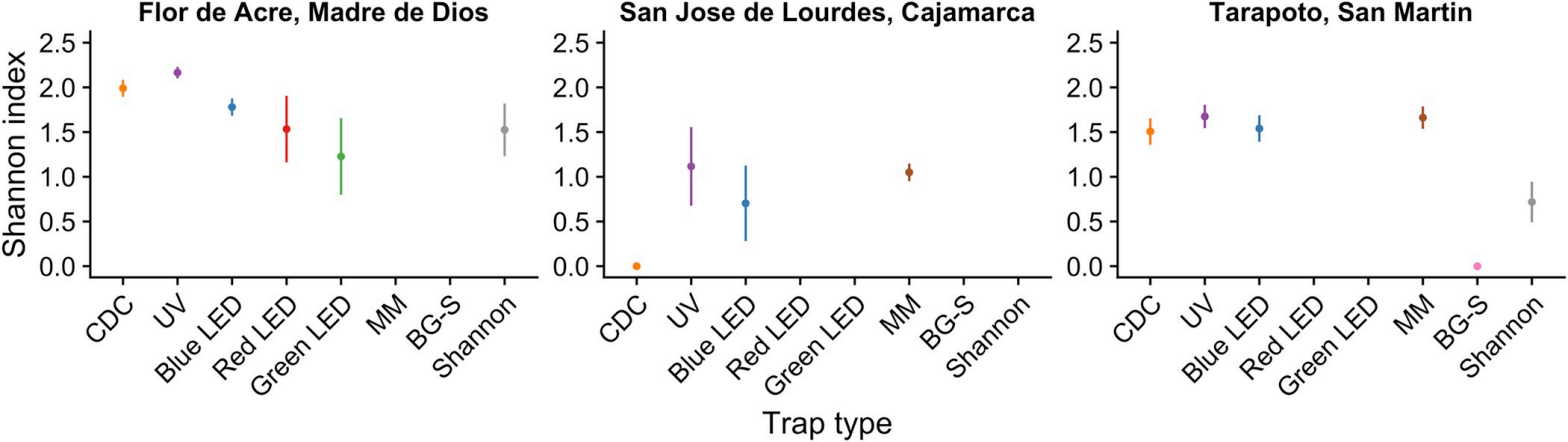
Comparison of sand fly Shannon diversity indices across trap types and study sites. The figure shows the Shannon diversity index (and estimated confidence intervals) for the different traps employed in collections. No panel was included for Caraz, Ancash because there were only two sand fly species trapped (S2 Fig).

The UV and blue LED traps collected the highest number of phlebotomine sand flies (53.4% and 21.3% respectively), while the green LED and red LED traps collected the lowest number (1.9% and 1.7% respectively) (S1 Table). To evaluate the effectiveness for sand fly collections of each trap type, the average of total sand fly specimens collected per hour per trap was calculated. Corrected for the total number of hours over two days of trapping, UV traps collected the greatest number of sand flies per hour (Fig 3). A Kruskal-Wallis test showed no significant differences in sand fly collections per trap type per day (chi-squared = 8.08, df = 5, p > 0.05), although there was large variation in sand fly trapping rates within each trap type over the two days of trapping (S1 Fig). Regarding species diversity index, UV traps collected a higher number of species richness (S2 Fig) and species evenness (Fig 4) than other trap types; the differences in Shannon diversity between UV traps and other traps at this site were all statistically significant (p < 0.05).

#### Tarapoto, San Martin

CDC, blue LED, UV, MM, BG-S, and Shannon trap were evaluated in three sites along Prolongación Alerta, Tarapoto district. A total of 1,335 phlebotomine sand flies were identified, 768 (57.5%) females and 567 (42.5%) males belonging to two genera, *Lutzomyia* (26 species) and *Brumptomyia* (2 species). Of them, 481 specimens were collected in the peridomicile and 854 inside the forest, but Kruskal-Wallis tests showed no significant differences in the sand fly species composition between both environments (p>0.05). *Lutzomyia hirsuta hirsuta* (SISA = 0.90), *Lu*. *nevesi* (SISA = 0.81) and *Lu*. (*Trichophoromyia*) spp. (SISA = 0.75) were the most abundant phlebotomine sand flies in all trap types used and all study sites. Regarding sand fly abundance by collection method, *Lu*. (*Trichophoromyia*) spp. was the predominant species in UV traps (49.3%), *Lu*. *nevesi* in blue LED (55.2%), CDC (57.6%) and MM (35.2%), *Lu*. *hirsuta hirsuta* in Shannon traps (80.2%) and MM (26.5%), and *Lu*. *yuilli yuilli* was caught principally with MM (98%) (S2 Table).

While the Shannon trap was used only on the first two days of trapping in favorable weather conditions, it captured the highest number of sand flies per hour compared to the other traps (Fig 3). During permanent rainy conditions on the last three days of trapping, UV traps performed better than the other four traps (S3 Fig). Kruskal-Wallis tests showed no significant differences in sand fly collections per trap type per day on the first two days with favorable weather (chi-squared = 5.8, df = 5, p > 0.05), on the last three days with permanent rain (chi-squared = 2.4, df = 3, p > 0.05), and across all five trapping days (chi-squared = 8.04, df = 5, p > 0.05). As with results from Madre de Dios, these results are explained by high variation in capture rates across trapping days within each trap type (S1 and S3 Figs). Shannon and BG-S traps captured a smaller number of sand fly species richness (S2 Fig) with significant lower species evenness compared to other trap types (p < 0.05; Fig 4).

#### San José de Lourdes, Cajamarca

Palmal and Nuevo Porvenir, two rural communities, were selected to evaluate MM, CDC, UV, blue LED, and BG-S traps. The rain was consistent during the entire sand fly collection period, so it was not possible to use the Shannon trap. A total of 191 phlebotomine sand flies were identified, 162 females (84.82%) and 29 males (15.18%) belonging to five *Lutzomyia* species. Of them, 171 specimens were collected in the peridomicile and 20 in edge of the forest, but Kruskal-Wallis tests showed no significant differences in the sand fly species composition between both environments (p>0.05). *Lu*. *maranonensis* (SISA = 0.94) and *Lu*. *robusta* (SISA = 0.56) were the most abundant. Other sand fly species identified were *Lu*. *pallidithorax*, *Lu*. *castanea* and *Lu*. *reclusa* (S3 Table).

The MM trap captured the greatest number of sand flies per hour compared to other trap types (Fig 3); BG-S traps did not capture any sand flies at this site. The Kruskal-Wallis test showed significant differences in sand fly collections per trap type per day (chi-squared = 13.2, df = 4, p < 0.05). However, the Wilcoxon signed-rank test with Benjamini-Hochberg FDR failed to detect significant differences (p > 0.05). The CDC trap captured a lower species richness compared to other trap types (S2 Fig) with significantly lower Shannon diversity (p < 0.05; Fig 4).

#### Caraz, Ancash

Choquechaca community, located 10 km from Caraz city in the inter-Andean Santa River valley, was the study site selected for evaluating CDC, UV, blue LED, and MM traps for sand fly collection. A total of 1,229 phlebotomine sand flies collected in peridomicile were identified, 1,170 females (95.2%) and 59 males (4.8%) belonging to two species, *Lu*. *verrucarum* (99.3%) and *Lu*. *peruensis* (0.7%) (S4 Table). Since there were only two species captured (S2 Fig) and a high predominance of *Lu*. *verrucarum*, Shannon diversity index was not calculated for this study site. MM traps captured the highest average number of sand fly specimens per hour and were the most effective traps (861 specimens, 70.1% of the total sand fly specimens), followed by CDC (189, 15.4%), UV (77, 6.3%), and blue LED (73, 5.9%) (Fig 3). Due to variation across trapping days (S1 Fig), no significant differences in sand fly collections per trap type were found by the Kruskal-Wallis test (chi-squared = 0.76, df = 3, p > 0.05).

### Detection of *Leishmania* DNA

#### kDNA PCR screening for *Leishmania*

A total of 353 pools of non-engorged female sand flies (1,613 specimens) were screened for *Leishmania* DNA (S5 Table). DNA of *Leishmania* parasites was detected in *Lu*. *hirsuta hirsuta* (1 pool), *Lu*. *davisi* (3 pools), *Lu*. *carrerai carrerai* (2 pools), *Lu*. *whitmani* (2 pools), and *Lu*. (*Trichophoromyia*) spp. (1 pool) from Madre de Dios collected with UV, blue LED, and green LED traps; and in *Lu*. *nevesi* (7 pools) *Lu*. *hirsuta hirsuta* (2 pools), *Lu*. (*Sciopemyia*) spp. (1 pool), and *Lu*. (*Pressatia*) spp. (1 pool) from San Martin collected with UV, blue LED, CDC, and MM traps (Table 2 and S5 Table). The estimated minimum infection rates using this molecular marker were 1.29% and 1.79% in Madre de Dios and San Martin sand flies, respectively. All analyzed sand fly pools from Cajamarca and Ancash were negative for *Leishmania* DNA.

**Table 2:** *Leishmania* DNA detection in phlebotomine sand flies from low jungle (Madre de Dios) and high jungle (San Martin) regions in Peru.

Region	Study site	Trap type	Trap code	*Lutzomyia* species	Females per pool	kDNA PCR	Nested *cytB* PCR	*cytB* sequencing
Madre de Dios	Flor de Acre	UV	TTE-005	*Lu*. (*Trichophoromyia*) spp.	10	+	-	
		UV	TTE-015	*Lu*. *hirsuta hirsuta*	1	+	-	
		UV	TTE-156	*Lu*. *carrerai carrerai*	3	+	+	*L*. *(V*.*) guyanensis*
		UV	TTE-157	*Lu*. *whitmani*	4	+	-	
		Blue LED	TTE-063	*Lu*. *carrerai carrerai*	6	+	-	
		Blue LED	TTE-133	*Lu*. *davisi*	10	+	-	
		Blue LED	TTE-140	*Lu*. *davisi*	10	+	-	
		Blue LED	TTE-144	*Lu*. *whitmani*	10	+	-	
		Green LED	TTE-036	*Lu*. *davisi*	2	+	-	
San Martin	Bocatoma de Shilcayo	MM	T14-SM-011	*Lu*. *hirsuta hirsuta*	9	+	-	
		Blue LED	T14-SM-127	*Lu*. *hirsuta hirsuta*	6	+	+	*L*. *(V*.*) naiffi*
	Centro de Rescate Urku	CDC	T14-SM-065	*Lu*. (*Sciopemyia*) spp.	1	+	-	
		CDC	T14-SM-146	*Lu*. *nevesi*	2	+	-	
		UV	T14-SM-160	*Lu*. *nevesi*	10	+	-	
		Blue LED	T14-SM-089	*Lu*. (*Pressatia*) spp.	1	+	-	
	Cordillera Escalera Lodge	CDC	T14-SM-079	*Lu*. *nevesi*	1	+	-	
		Blue LED	T14-SM-119	*Lu*. *nevesi*	10	+	-	
		Blue LED	T14-SM-120	*Lu*. *nevesi*	10	+	-	
		Blue LED	T14-SM-121	*Lu*. *nevesi*	10	+	-	
		Blue LED	T14-SM-123	*Lu*. *nevesi*	10	+	-	

#### Nested *cytB* PCR and DNA sequencing for *Leishmania*

**Fig 5:**
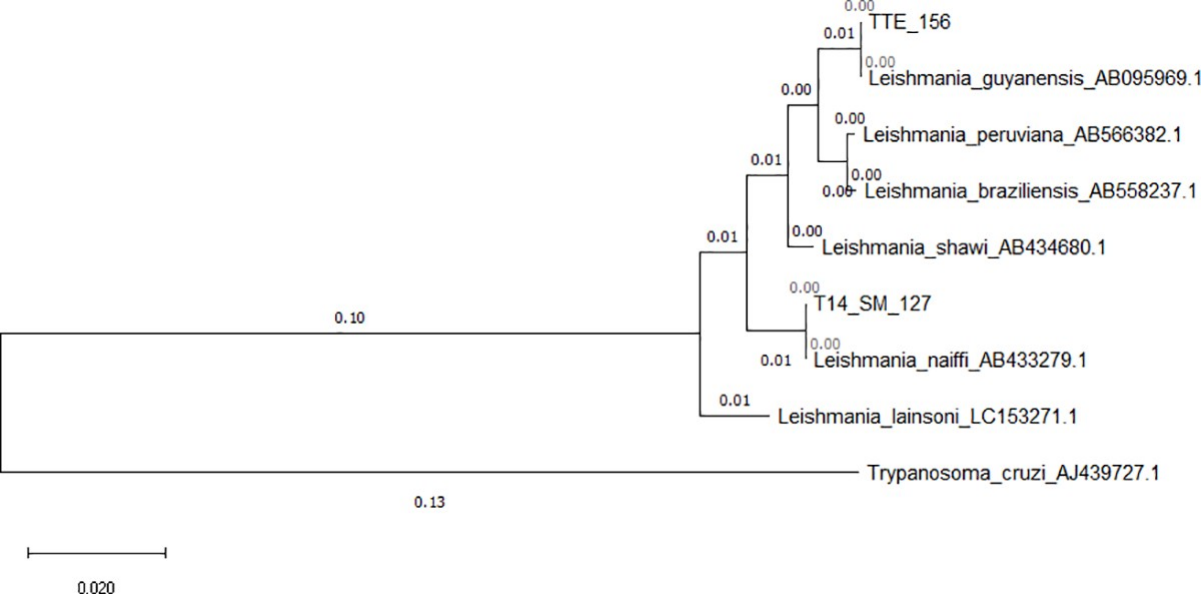
Maximum likelihood tree for *Leishmania* species in *Lutzomyia* spp. based on cytochrome b (*cytB*) sequences. TTE-156 = *Lu*. *carrerai carrerai* from Madre de Dios (low jungle) and T14-SM127 = *Lu*. *hirsuta hirsuta* from San Martin (high jungle). The numbers on the branches represent bootstrap support and the size bar indicates 0.02 substitutions per site. The tree was rooted with the sequence of *Trypanosoma cruzi*.

Nested *cytB* PCR amplicons from positive pools were sequenced. One pool of *Lu*. *carrerai carrerai* collected with a UV trap from Madre de Dios was found infected with *L*. *(V*.*) guyanensis;* one pool of *Lu*. *hirsuta hirsuta* collected with a blue LED trap from San Martin were found infected with *L*. *(V*.*) naiffi* (Table 2). The phylogenetic tree with *cytB* DNA sequences is shown in Fig 5. The estimated minimum infection rates using this molecular marker were 0.13% and 0.16% in Madre de Dios and San Martin sand flies, respectively.

### Detection of *Bartonella* DNA

#### ITS PCR and nested *gltA*, *rpoB* and *ftsZ* PCR screening for *Bartonella*

A total of 282 pools of non-engorged female sand flies (1,450 specimens) were screened for *Bartonella* DNA (S6 Table). *Lu*. *whitmani* (1 pool) from Madre de Dios collected with UV trap; *Lu*. *nevesi* (12 pools), *Lu*. *hirsuta hirsuta* (2 pools) and *Lu*. *sherlocki* (1 pool) from San Martin collected with CDC, blue LED, UV, and MM traps; and *Lu*. *maranonensis* (1 pool) from Cajamarca collected with blue LED were found infected with *Bartonella* spp. by ITS PCR, and confirmed by nested *gltA* PCR, and additional molecular markers *rpoB* and *ftsZ* PCR (Table 3 and S6 Table). The minimum infection rate based on positive *gltA* PCR was 1.02%, 1.29% and 0.65% for Madre de Dios, San Martin, and Cajamarca respectively. No positive pools were found in non-engorged female sand flies from Ancash.

**Table 3:** *Bartonella* DNA detection in phlebotomine sand flies from low jungle (Madre de* Dios) and high jungle (San Martin and Cajamarca) regions in Peru.

Region	Study stie	Trap type	Trap code	*Lutzomyia* species	Females per pool	ITS PCR	Nested *gltA* PCR	Nested *rpoB* PCR	Nested *ftsZ* PCR	*gltA* sequencing
Madre de Dios	Flor de Acre	UV	TTE-006	*Lu*. *whitmani*	10	+	+	+	-	*B*. *bacilliformis*-like
San Martin	Bocatoma de Shilcayo	MM	T14-SM-011	*Lu*. *hirsuta hirsuta*	9	+	+	+	-	*B*. *bacilliformis*-like
		CDC	T14-SM-132	*Lu*. *hirsuta hirsuta*	8	+	+	+	-	*B*. *bacilliformis*-like
	Centro de Rescate Urku	CDC	T14-SM-046	*Lu*. *nevesi*	10	+	+	+	-	*B*. *bacilliformis*-like
		CDC	T14-SM-047	*Lu*. *nevesi*	10	+	+	+	-	*B*. *bacilliformis*-like
		CDC	T14-SM-060	*Lu*. *nevesi*	10	+	+	+	-	*B*. *bacilliformis*-like
		CDC	T14-SM-145	*Lu*. *nevesi*	10	+	+	+	-	*B*. *bacilliformis*-like
		UV	T14-SM-160	*Lu*. *nevesi*	10	+	+	+	-	*B*. *bacilliformis*-like
		UV	T14-SM-161	*Lu*. *nevesi*	10	+	+	-	-	*B*. *bacilliformis*-like
		CDC	T14-SM-038	*Lu*. *nevesi*	1	+	+	+	+	*Candidatus* B. rondoniensis-like
	Cordillera Escalera Lodge	UV	T14-SM-052	*Lu*. *sherlocki*	1	+	+	+	-	*B*. *bacilliformis*-like
		Blue LED	T14-SM-115	*Lu*. *nevesi*	10	+	+	+	-	*B*. *bacilliformis*-like
		Blue LED	T14-SM-119	*Lu*. *nevesi*	10	+	+	-	-	*B*. *bacilliformis*-like
		Blue LED	T14-SM-120	*Lu*. *nevesi*	10	+	+	+	-	*B*. *bacilliformis*-like
		Blue LED	T14-SM-122	*Lu*. *nevesi*	10	+	+	+	-	*B*. *bacilliformis*-like
		Blue LED	T14-SM-123	*Lu*. *nevesi*	10	+	+	+	-	*B*. *bacilliformis*-like
Cajamarca	San José de Lourdes	Blue LED	T14-SJ-144	*Lu*. *maranonensis*	4	+	+	+	+	*Candidatus* B. rondoniensis-like

#### *Bartonella* DNA sequencing

**Fig 6:**
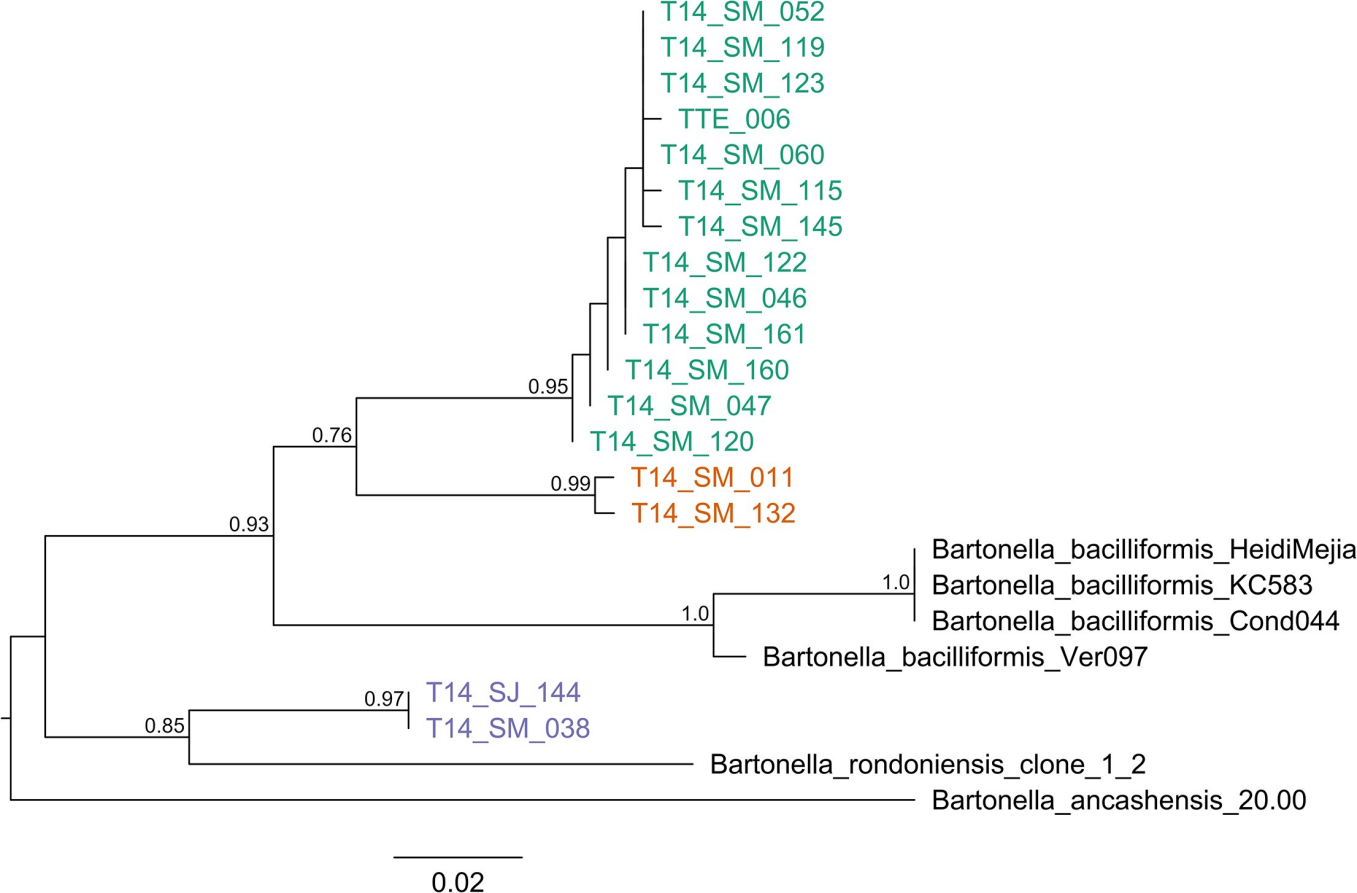
Maximum likelihood tree for *Bartonella* species in *Lutzomyia* spp. based on *gltA* gene sequences. The three lineages are in colors, lineage 1 in green, lineage 2 in orange, and lineage 3 in purple. Lineages 1 and 2 are related to *B*. *bacilliformis*, and lineage 3 is related to *Candidatus* B. rondoniensis. TTE-006 = *Lu*. *whitmani* from Iberia, Madre de Dios; T14-SM-011 and T14-SM-132 = *Lu*. *hirsuta hirsuta* from San Martin; T14-SM-052 = *Lu*. *sherlocki* from San Martin; T14-SM-038, T14-SM-046, T14-SM-047, T14-SM-060, T14-SM-115, T14-SM-119, T14-SM-120, T14-SM-122, T14-SM-123, T14-SM-145, T14-SM-160, T14-SM-161 = *Lu*. *nevesi* from San Martin; and T14-SJ-144 = *Lu*. *maranonensis* from Cajamarca. The numbers on the branches represent bootstrap support and the size bar indicates 0.02 substitutions per site.

Amplicons from all positive pools by ITS and *gltA* were sequenced and confirmed as *Bartonella* DNA using BLAST. ITS sequences varied in length between 461–590 bp and the closest matching *Bartonella* species on GenBank were *B*. *bacilliformis* (average sequence identity = 91.5%) and *B*. *ancashensis* (average sequence identity = 88.1%). The presence of insertions and deletions in the alignment of ITS sequences complicates phylogenetic analyses, so phylogenetic trees were constructed only for the *gltA* gene. The DNA sequences of *gltA* had lengths between 319–366 bp and were classified into three lineages (Fig 6). Lineage 1 comprised *Lu*. *whitmani* (1 pool) from Madre de Dios, and *Lu*. *nevesi* (11 pools) and *Lu*. *sherlocki* (1 pool) from San Martin, which were found infected with genotypes most closely related to *B*. *bacilliformis* (average sequence identity = 89.6%). Lineage 2 comprised two pools of *Lu*. *hirsuta hirsuta* from San Martin and were also related to *B*. *bacilliformis* (average sequence identity = 89.6%). The third lineage comprised of one pool of *Lu*. *nevesi* from San Martin and one pool of *Lu*. *maranonensis* from Cajamarca. These species were found infected with a genotype most closely related to *Candidatus* B. rondoniensis (average sequence identity = 92%).

## Discussion

This is the first study in Peru to evaluate the efficacy of different light and odor-baited trap types for phlebotomine sand fly collection in different ecological regions, and is the first study to determine the natural infection of *Leishmania* spp. and *Bartonella* spp. in multiple sand flies in different endemic areas outside of the Andean region. Both findings are unique in Peru and will help to better understand the behavior of phlebotomine sand fly vectors.

Knowledge about insect color vision suggests that there is a high sensitivity to light with short wavelengths that correspond to the ultraviolet spectrum in most insect families [68]. However, due to the higher diversity of visual conditions in which insects operate in nature and the higher diversity of habitats they have colonized, the sensitivity and response to spectral light at different intensities and wavelengths can vary across sites and among species, as has been reported previously [42,68,69]. In the Peruvian southeastern low jungle area (Madre de Dios region), where previous research has reported a great diversity and abundance of phlebotomine sand flies (>60 species), we have observed that the sand fly response of phlebotomine sand flies to light is in decreasing order at this site: UV > blue LED > incandescent light (CDC) > green LED > red LED (S1 Fig). The finding of a higher response to UV light in sand fly species from the Peruvian southeastern region is comparable to results obtained in Iraq and Italy [4,70]. The response of phlebotomine sand flies to green and red LED in this study was poor (1.7% of the total sand flies collected) in contrast to results obtained in Egypt [31], where the red LED was the most efficient trap type for catching *Phlebotomus papatasi* and results in the Brazilian rainforest [71,72], where they found that the green LED trap was the most effective in comparison to blue LED and CDC control (38–42% of the total sand flies collected).

Except for Shannon traps and protected human bait, traps that release CO_2_ and other chemical attractants are very effective for collecting phlebotomine sand flies and other blood sucking insects as has been observed in Israel and Egypt [27,32], where different commercial models of MM releasing CO_2_ only successfully captured *P. papatasi* and other sand fly species. Most studies with MM traps have been for surveillance of mosquito vectors of malaria and arboviruses [73,74] and experimental vector control programs [75]. For *Aedes* and other mosquito species, MM trap efficacy is increased when in addition to CO_2_ the trap is supplemented with octenol [76]. However, when MM was compared with other traps, their efficacy was variable. Human Landing Collection (HLC) was more efficient for collection of *Anopheles* species (68.13%) compared with MM (31.88%) [34,77]; otherwise, MM collected more abundant specimens of Culicidae (59.94%) than CDC light traps (40.06%) [74]. In our study, the MM was the most efficient sand fly collection method in high jungle areas of Cajamarca region, when under unfavorable weather conditions with permanent rain, more than 80% of phlebotomine sand flies were collected with this trap type (Fig 3). Similar effectiveness of MM for sand fly collections was observed in inter-Andean valleys of Caraz, Ancash (Fig 3).

Our results of the trap type evaluation conducted in different ecological regions with distinct sand fly species composition indicate that phlebotomine sand fly behavior against different colors of light or chemical attractants is variable between study sites. In low jungle regions, UV and blue LED were more efficient and collected a higher diversity of sand fly species, whereas in high jungle and inter-Andean regions, MM and UV traps were more effective. In general, in our study we have observed that traps that use short wavelength light collect a higher diversity of species (Fig 4), while the traps that use chemical attractants as CO_2_ and R-Octenol, are more selective to capture anthropophilic sand fly species, so it is recommended to use a combination of both trap types (traps based on light emission combined with traps that use chemical attractants) in entomological surveillance of potential sand fly vectors of leishmaniasis and bartonellosis.

It is known that leishmaniasis has been endemic in the Peruvian southeastern region for a long time [78], but the potential vectors have not been characterized until recent years. In communities located near the triple border between Peru, Brazil and Bolivia, 16 *Lutzomyia* species were found naturally infected with *Leishmania* parasites [37,44]. In the current study, we found *Lu*. *carrerai carrerai* naturally infected with *L*. (*V*.) *guyanensis* in Iberia, Madre de Dios. This *Leishmania* species has a wide distribution in the Amazon jungle, and in Peru it has been isolated from patients with cutaneous leishmaniasis from high and low jungle regions [79,80]. At the same study site, *Lu*. *whitmani* was found naturally infected with a genotype of *Bartonella* spp. (lineage 1) most closely related to *B*. *bacilliformis* based on *gltA* gene DNA sequences. There are no reports of autochthonous Carrion’s disease transmission in Iberia or any other sites in Madre de Dios region. Recently was reported the detection of *B*. *bacilliformis* in ticks collected from wild mammals in San Lorenzo [81], a small town located along the interoceanic highway in Madre de Dios, using a real time PCR protocol, but these results are doubtful because they were not supported by DNA sequencing and phylogenetic analysis [82,83]. Currently, all efforts to find *B*. *bacilliformis* in other mammalian reservoirs other than humans have failed, as well as any incrimination of other arthropod vectors besides *Lutzomyia* species [14,82–85].

In San Martin, northern Amazonian region, where leishmaniasis has higher prevalence and is an important risk area for transmission of this disease [6,86], we found *Lu*. *hirsuta hirsuta* naturally infected with *L*. (*V*.) *naiffi* in a rural area located only 5 kilometers from Tarapoto city, along the road to Cordillera Escalera. This is the first report of *L*. (*V*.) *naiffi* in Peru, which increases the number of *Leishmania* species in our country to eight, in addition to *L*. *(V*.*) braziliensis*, *L*. (*V*.) *peruviana*, *L*. (*V*.) *guyanensis*, *L*. (*V*.) *lainsoni*, *L*. (*V*.) *shawi*, *L*. *(L*.*) amazonensis*, and a hybrid *L*. *braziliensis*/*peruviana* [80]. *Leishmania naiffi* causes cutaneous leishmaniasis and is widespread in the Brazilian Amazonian region [87] and has also been found infecting human patients as well as *Lu*. *tortura* in the northern Amazonian region of Ecuador [50]. Our results suggest that *L*. (*V*.) *naiffi* has a wide distribution in the Amazon region and could be responsible for leishmaniasis cases in San Martin department, where *L*. (*V*.) *guyanensis* has also been detected in human patients [79,80]. Further studies on human populations are necessary to confirm *L*. (*V*.) *naiffi* transmission in Peru. In our study sites in San Martin we also found *Lu*. *nevesi*, *Lu*. *hirsuta hirsuta* and *Lu*. *sherlocki* naturally infected with two *Bartonella* genotypes related to *B*. *bacilliformis* based on *gltA* gene sequencing (lineages 1 and 2), and a third genotype of *Bartonella* spp. related to *Candidatus* B. rondoniensis naturally infecting *Lu*. *nevesi* (lineage 3). This is the first report of detection of *Bartonella* DNA in *Lutzomyia* species from the northern Amazonian region of San Martin. Leishmaniasis cases have a high prevalence in San Martin and are reported yearly by the Ministry of Health, but information about human bartonellosis in this region is very limited, with only a few cases reported during the last years but without evidence of autochthonous transmission [88]. Interestingly, we found that all sand fly species infected with *Bartonella* and *Leishmania* in Madre de Dios and San Martin regions had previously been reported as potential leishmaniasis vectors in the Amazon region of Peru and other countries [3,44].

The *Bartonella* DNA genotype related to *Candidatus* B. rondoniensis (lineage 3) was also found naturally infecting *Lu*. *maranonensis* in San Jose de Lourdes, a high jungle area in Cajamarca region. This sand fly species was recently found infected with *B*. *bacilliformis* in an endemic area of Carrion’s disease in Cutervo province (Cajamarca central region) and has been incriminated as a potential vector of this disease [41]. *Lutzomyia maranonensis* and *Lu*. *robusta* are the most abundant phlebotomine sand flies in the Peruvian northeastern region and southern Ecuador (Zamora Chinchipe province), and it has been suggested that these sand fly species could be the vectors of Carrion’s disease and leishmaniasis in the Peru-Ecuador border regions [19,89,90]. Our results and those of Ulloa *et al*. [41] show that *Lu*. *maranonensis* is capable of harboring different *Bartonella* species. Similar results were reported by Villaseca *et al*. [38] who found *Lu*. *peruensis* naturally infected with *B*. *bacilliformis* and *B*. *taylori* in the Urubamba valley, Cuzco region, Peru, where an important outbreak of acute Carrion’s disease occurred during 1997–1998 without previous history of this disease in this region [91]. *Candidatus* B. rondoniensis was first found in triatomine bugs vectors of Chagas disease, *Eratyrus mucronatus* from French Guiana [92]. In Peru, there are no reports of *Bartonella* species infecting triatomine bugs, but different species of Triatominae are distributed across the country, especially in the northern region (San Martin, Cajamarca and Amazonas regions) where Chagas disease is endemic [93–96]. Further studies are needed to determine if triatomine bugs in Peru are also infected with *Bartonella* spp.

Regarding sand fly species found naturally infected with *Bartonella* spp. in this study, the majority of them are recognized as potential vectors of leishmaniasis, including *Lu*. *whitmani*, *Lu*. *nevesi* and *Lu*. *sherlocki* [44], whereas *Lu*. *maranonensis* is a suspected vector of leishmaniasis along the Peru-Ecuador border [90]. Our detection of *Bartonella* DNA related to *B*. *bacilliformis* in sand flies was unexpected. Due the high diversity of *Bartonella* species in different vertebrate hosts such as bats, rodents, cats, dogs, non-human primates and other wild vertebrates [91,97–101], and arthropod vectors such as ticks, fleas, body lice, biting flies and triatomine bugs [11,12,102,103], it is unclear which hosts are reservoirs for *B*. *bacilliformis*-like lineages 1 and 2 or *Candidatus* B. rondoniensis-like lineage 3. While the lineages were most closely related to *B*. *bacilliformis* and *Candidatus* B. rondoniensis, they are still quite distant from these species and each other in absolute genetic relatedness (<95% sequence similarity) and may thus represent novel *Bartonella* species [104]. However, additional markers beyond *gltA* must be used to clarify the phylogenetic associations of these lineages with other *Bartonella* species [55,83,104]. Our data and that from Laroche *et al*. [92] with the description of *Candidatus* B. rondoniensis indicate that there is an underappreciated diversity of *Bartonella* lineages present in South American sand flies and other insects that may give us deeper insight into the origins of *B*. *bacilliformis*. Finally, the role of these *Bartonella* species in human disease is unknown. Further studies are needed to determine if these *Bartonella* genotypes detected in sand flies from low jungle (Madre de Dios) and high jungle (San Martin and Cajamarca) regions have the ability to cause human disease, or only infect vertebrate hosts.

According to our trap type evaluation in endemic areas of leishmaniasis and bartonellosis in Peru, the effectiveness of different traps for collecting sand flies vary according to ecological characteristics, climatic conditions and sand fly fauna composition across each site. Therefore, we recommend combining different traps to efficiently survey sand fly diversity across multiple habitats and maximize the surveillance of pathogens they may carry. Regarding pathogen detection in sand flies, this is the first report of *L*. *(V*.*) naiffi* in Peru and of detection of *Bartonella* spp. genotypes distantly related to *B*. *bacilliformis* and *Candidatus* B. rondoniensis infecting sand fly species from the Amazon jungle. Our results underscore the need for increased studies of the ecological aspects of Amazonian sand fly species, and to increase Carrion’s disease surveillance in non-endemic areas, especially in the Amazon jungle where the risk of transmission for this disease can be higher due the capacity of sand fly species to transmit different human and newly emerging pathogens.

## Supporting information

S1 Fig Comparison of sand flies per hour across trap types and study sites for each sampling day. (TIF)

S2 Fig Comparison of sand fly species richness across trap types and study sites. (TIF)

S3 Fig Comparison of sand flies per hour versus trap type in Tarapoto, San Martin, on days with good weather and permanent rain: (A) cumulative sand flies per hour; (B) sand flies per hour on separate trapping days.
(TIF)

S1 Table Sand fly species (females and males) identified by collection trap from Flor de Acre, Iberia, Madre de Dios in 2014. (XLSX)

S2 Table Sand fly species (females and males) identified by collection trap from Tarapoto, San Martin in 2015. (XLSX)

S3 Table Sand fly species (females and males) identified by collection trap from San Jose de Lourdes, Cajamarca in 2015. (XLSX)

S4 Table Sand fly species (females and males) identified by collection trap from Choquechaca, Caraz, Ancash in 2015. (XLSX)

S5 Table *Leishmania* DNA screening in phlebotomine sand flies by species, females per pool, locality, and collection trap for all regions by kDNA PCR and nested cytB PCR. (XLSX)

S6 Table *Bartonella* DNA screening in phlebotomine sand flies by species, females per pool, locality, and collection trap for all regions by ITS PCR and nested gltA PCR. (XLSX)

S1 Text Molecular screening of *Leishmania* and *Bartonella* DNA in sand flies. (DOCX)
