# Supplementary figures and images for: Comparison of sand fly trapping approaches for vector surveillance of Leishmania and Bartonella species in ecologically distinct, endemic regions of Peru
Source: PLoS Negl Trop Dis. 2021 Jul 14;15(7):e0009517. doi: 10.1371/journal.pntd.0009517 (PMC8279425; doi:10.1371/journal.pntd.0009517)

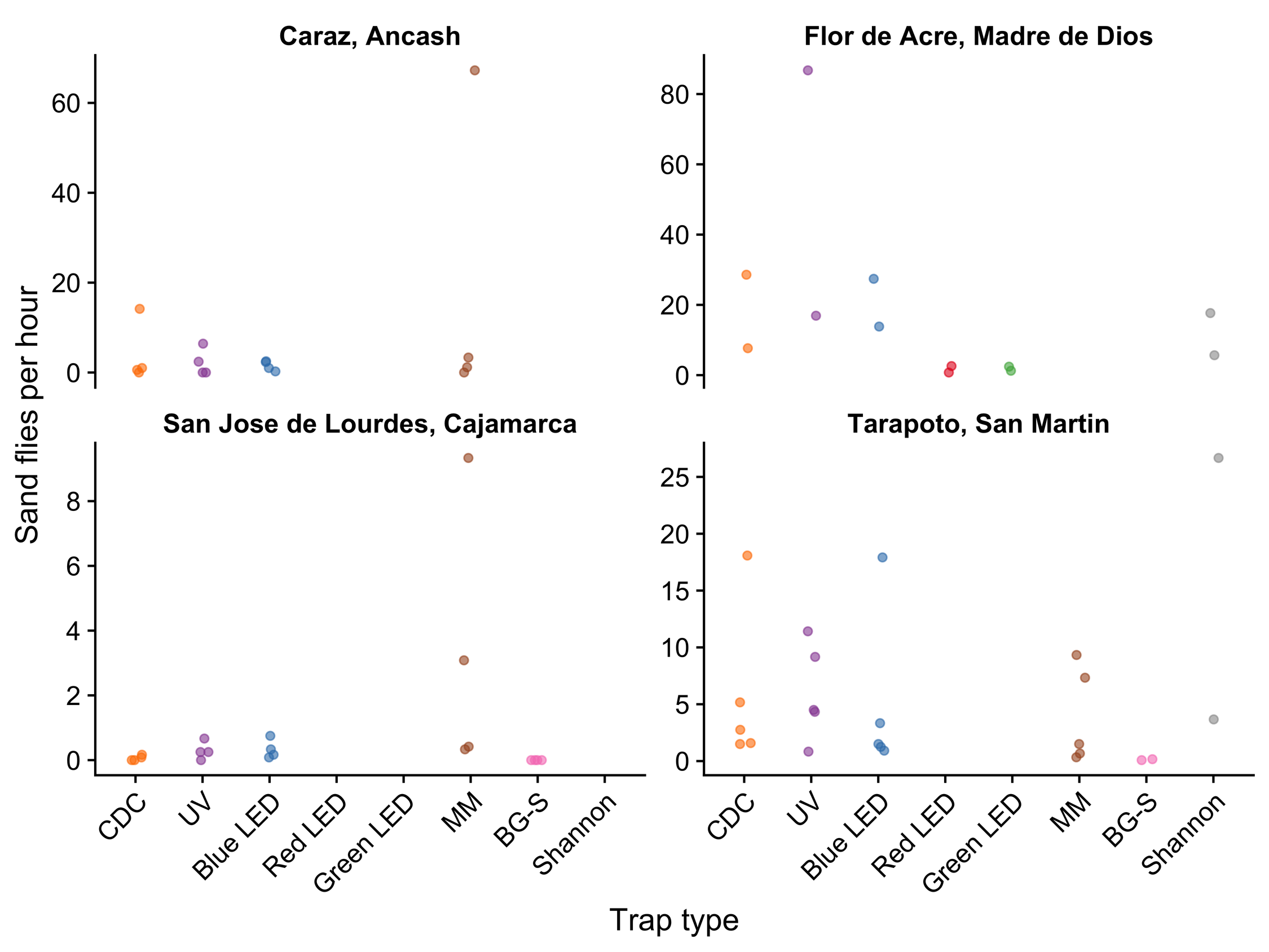

Supplement: S1 Fig — (TIF) [file pntd.0009517.s001.tif]

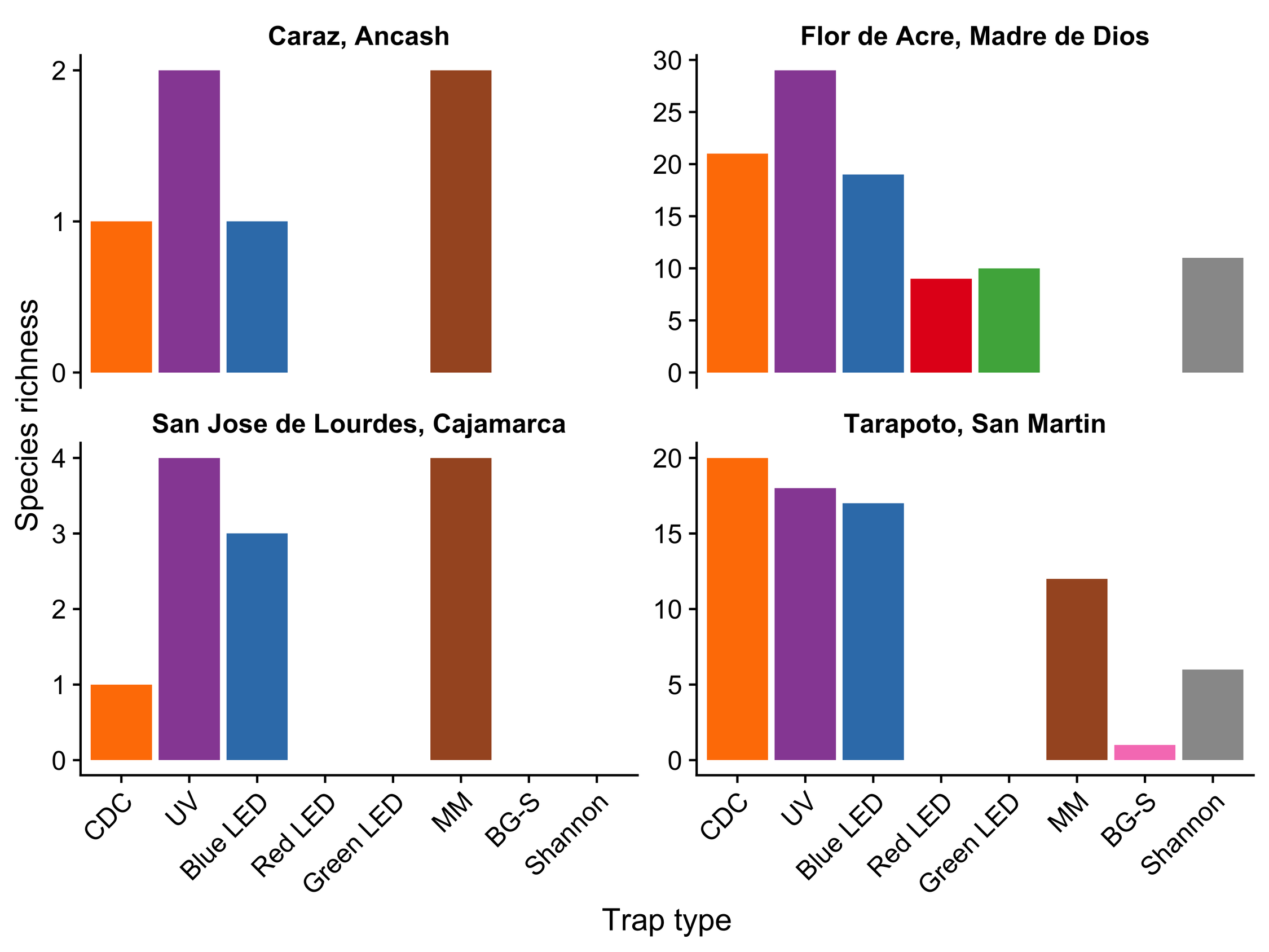

Supplement: S2 Fig — (TIF) [file pntd.0009517.s002.tif]

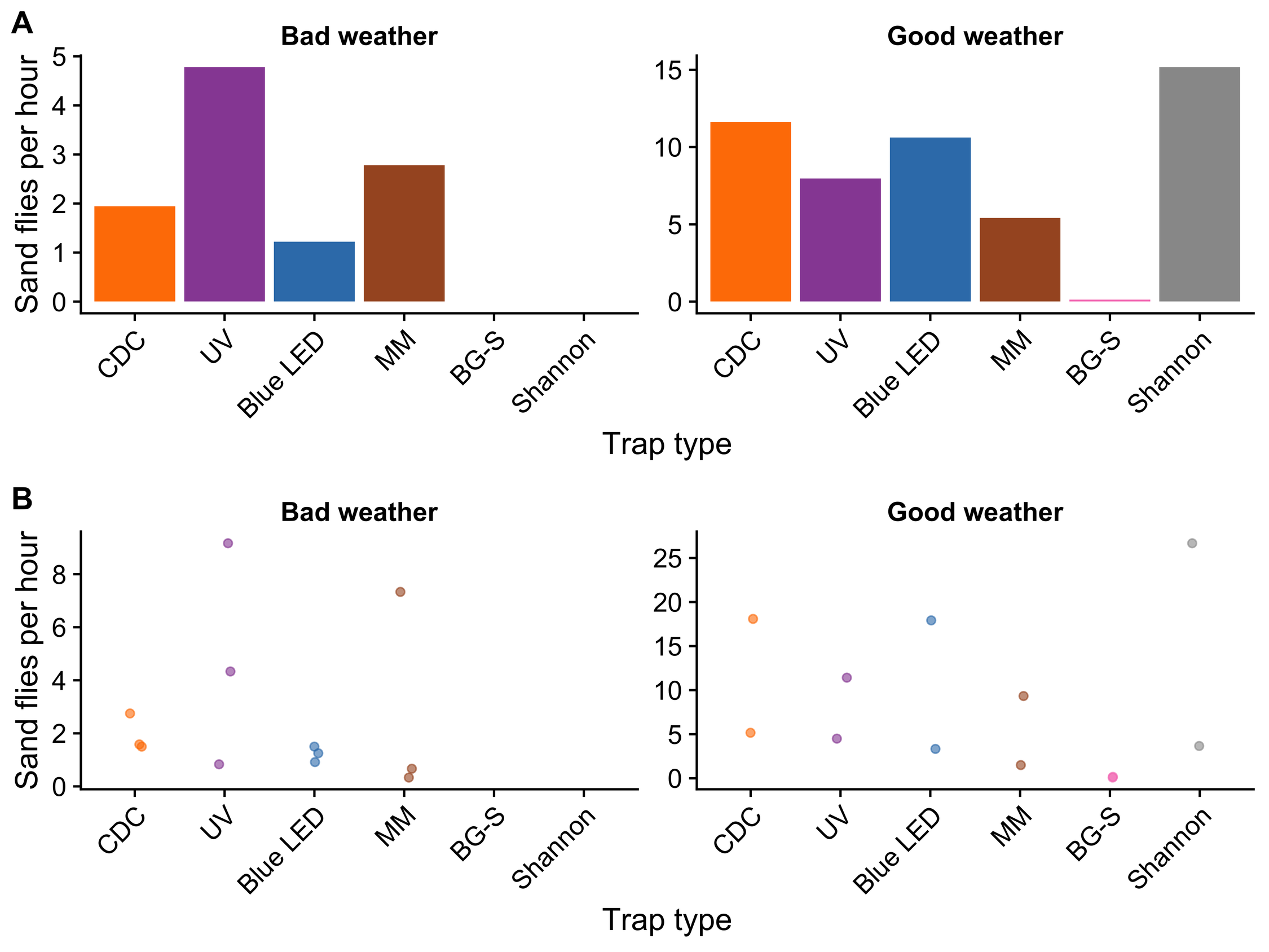

Supplement: S3 Fig — Comparison of sand flies per hour versus trap type in Tarapoto, San Martin, on days with good weather and permanent rain: (A) cumulative sand flies per hour; (B) sand flies per hour on separate trapping days. (TIF) [file pntd.0009517.s003.tif]
