# Supplementary material for: Comparison of sand fly trapping approaches for vector surveillance of Leishmania and Bartonella species in ecologically distinct, endemic regions of Peru
Source: PLoS Negl Trop Dis. 2021 Jul 14;15(7):e0009517. doi: 10.1371/journal.pntd.0009517 (PMC8279425; doi:10.1371/journal.pntd.0009517)
Supplement: S1 Text — (DOCX) [file pntd.0009517.s010.docx]

**Molecular screening of *Leishmania* and *Bartonella* DNA in sand flies**

**Polymerase chain reaction for detection of *Leishmania* DNA**. *Leishmania* DNA was detected by kDNA PCR that targets *Leishmania* minicircle kinetoplast DNA sequences (approx. 700bp) conserved among species, using primers L.MC-1S and L.MC-1R at a 0.4µM final concentration [1]. The conventional PCR reaction was carried out in a final volume of 25 µL with 1µL of DNA template, using MgCl_2_ at a 1.5mM final concentration, dNTPs at a 0.2mM final concentration and 1.25 units of GoTaq Flexi DNA polymerase (Promega, Madison, WI, USA). After an initial denaturation at 95°C for 5 minutes, the PCR amplification was performed with 30 cycles of denaturation (95°C for one minute), annealing (55°C for one minute), and polymerization (72°C for one minute), with a final extension at 72°C for 10 minutes [1]. *Leishmania* species were identified using the partial cytochrome *b* gene (*cytB*; between 730-850bp) amplified with a nested PCR [2,3]. The first PCR amplification was performed using L.cyt-AS and L.cyt-AR primers and 2µL of DNA. The second PCR amplification was performed using L.cyt-S and L.cyt-R primers and 1µL of the first PCR. PCR conditions of the *cytB* nested PCR were the same as the kDNA PCR.

**Polymerase chain reaction for detection of *Bartonella* DNA.** A PCR that amplifies a segment between 400-600bp of the 16S–23S internal transcribed spacer (ITS) region was used for sand fly screening [4,5]. The PCR was carried out in a volume of 25 µL with 5 µL of DNA template, using ITS 325S and ITS 1100AS primers at a 0.4 µM final concentration and Platinum High Fidelity Master Mix PCR solution (Invitrogen, Carlsbad, CA, USA). After initial denaturation at 95°C for 3 minutes, the PCR was performed with 55 cycles of denaturation (95°C for 30 seconds), annealing (66°C for 30 seconds), and polymerization (72°C for 30 seconds), and final extension at 72°C for 7 minutes. To identify *Bartonella* species, the ITS-positive samples were analyzed by three molecular markers: the citrate synthase gene (*gltA*), the β subunit of the RNA polymerase gene (*rpoB*), and the filamenting temperature-sensitive mutant Z protein gene (*ftsZ*). Nested PCR for the three partial genes were performed using High Fidelity Master Mix PCR solution (Invitrogen). The first amplification of the *gltA* nested PCR was performed using 443F and 1210R primers at a 0.4 µM final concentration [6,7] with the following PCR conditions: initial denaturation at 95°C for 5 minutes followed by 20 cycles of denaturation (95°C for 30 seconds), annealing (48°C for 30 seconds), and polymerization (72°C for 2 minutes), and final extension at 72°C for 7 minutes. The Bhcs.781p and Bhcs.1137n primers at a 0.4 µM final concentration [8] were used in the second *gltA* PCR amplification with the following conditions: initial denaturation at 95°C for 5 minutes followed by 40 cycles of denaturation (95°C for 30 seconds), annealing (55°C for 30 seconds), and polymerization (72°C for 1 minute), and final extension at 72°C for 7 minutes. The first amplification of the *rpoB* nested PCR was performed using 1350f and 2350r primers at a 0.4 µM final concentration [9] with the following PCR conditions: initial denaturation at 95°C for 4 minutes followed by 40 cycles of denaturation (95°C for 30 seconds), annealing (48°C for 60 seconds), and polymerization (72°C for 2 minutes), and final extension at 72°C for 10 minutes. The second *rpoB* PCR round was performed with primers 1400f and 2300r with the following PCR conditions: initial denaturation at 95°C for 4 minutes followed by 35 cycles of denaturation (95°C for 30 seconds), annealing (50°C for 30 seconds), and polymerization (72°C for 1 minute), and final extension at 72°C for 10 minutes [10]. The first round of the *ftsZ* nested PCR was performed with primers Bfp1 and Bfp2 [11] with the following PCR conditions: initial denaturation at 95°C for 4 minutes followed by 40 cycles of denaturation (95°C for 30 seconds), annealing (55°C for 30 seconds), and polymerization (72°C for 1 minute), and final extension at 72°C for 10 minutes. The second *ftsZ* round was performed with primers R83 and L83 [12] with the following PCR conditions: initial denaturation at 95°C for 4 minutes followed by 40 cycles of denaturation (95°C for 30 seconds), annealing (54°C for 30 seconds), and polymerization (72°C for 1 minute), and final extension at 72°C for 10 minutes.

**References**

1. Kato H, Uezato H, Katakura K, Calvopiña M, Marco J, Barroso P *et al.* Detection and identification of *Leishmania* species within naturally infected sand flies in the Andean areas of Leishmaniasis in Ecuador by a Polymerase Chain Reaction. Am J Trop Med Hyg. 2005; 72(1):87–93. <http://citeseerx.ist.psu.edu/viewdoc/download?doi=10.1.1.484.1044&rep=rep1&type=pdf>
2. Kato H, Caceres AG, Mimori T, Ishimaru Y, Sayed AS, Fujita M *et al.* Use of FTA cards for direct sampling of patients' lesions in the ecological study of cutaneous leishmaniasis. J Clin Microbiol. 2010; 48(10):3661-5. Epub 2010/08/20. https://doi.org/10.1128/JCM.00498-10 PMID: 20720027; PubMed Central PMCID: PMC2953078.
3. Kato H, Calvopiña M, Criollo H, Hashiguchi Y. First human cases of *Leishmania (Viannia) naiffi* infection in Ecuador and identification of its suspected vector species. Acta Tropica. 2013; 128: 710-713.

Diniz, P.P., Maggi, R.G., Schwartz, D.S., Cadenas, M.B., Bradley, J.M., Hegarty, B.C., Breitschwerdt, E.B. Canine bartonellosis: serological and molecular prevalence in Brazil and evidence of co-infection with *Bartonella henselae* and *Bartonella vinsonii* subsp. *berkhoffii*. Vet. Res. 2007; 38, 697–710. <https://doi.org/10.1051/vetres:2007023>

1. Billeter S, Caceres A, Gonzales J, Luna D, Kosoy M. Molecular detection of *Bartonella* species in ticks from Peru. J Med Entomol. 2011; 48(6):1257-60. <https://www.ncbi.nlm.nih.gov/pubmed/22238888>
2. Birtles R, Raoult D. Comparison of partial *Citrate Synthase Gene* *(gltA)* sequences for phylogenetic analysis of *Bartonella* species. Int J Syst Bacteriol. 1996; 46(4): 891-897.
3. Gundi V, Kosoy M, Makundi R, Laudisoit A. Identification of diverse *Bartonella* genotypes among small mammals from Democratic Republic of Congo and Tanzania. Am J Trop Med Hyg. 2012; 87(2): 319-326.
4. Norman A, Regnery R, Jameson P, Greene C, Krause D. Differentiation of *Bartonella*-like isolates at the species level by PCR-Restriction Fragment length polymorphism in the Citrate Synthase Gene. J Clin Microbiol. 1995; 33(7):1797-1803
5. Kabeya H, Inoue K, Izumi Y, Morita T, Imai S, Maruyama S. *Bartonella* Species in Wild Rodents and Fleas from Them in Japan. J Vet Med Sci. 2011; 73. 1561-7. 10.1292/jvms.11-0134. doi: 10.1292/jvms.11-0134. Epub 2011 Jul 27.
6. Renesto P, Gouvernet J, Drancourt M, Roux V, Raoult D. Use of *rpoB* gene analysis for detection and identification of *Bartonella* species. J Clin Microbiol. 2001; 39(2): 430-437.
7. Zeaiter Z, Liang Z, Raoult D. Genetic classification and differentiation of *Bartonella* species based on comparison of partial ftsZ gene sequences. J Clin Microbiol. 2002; 40(10): 3641-3647.
8. Colborn J, Kosoy M, Motin V, Telepnev M, Valbuena G, Myint K *et al.* Improved detection of *Bartonella* DNA in mammalian hosts and arthropod vectors by Real-Time PCR using the NADH Dehydrogenase Gamma Subunit (*NuoG*). J Clin Microbiol. 2010; 48(12): 4630-4633. DOI:10.1128/JCM.00470-10.
